# Accuracy of a Novel Desktop Micro-CT Scanner for Direct Digitization of Dental Impressions: A Comparative In Vitro Study

**DOI:** 10.3390/dj14010016

**Published:** 2026-01-01

**Authors:** Jiaying Gu, Liqing Zhu, Wenyue Yang, Yuan Zhang, Fan He, Yunwen Xu, Xiaoyu Gu, James Kit Hon Tsoi, Yuanfei Fu

**Affiliations:** 1 Department of Prosthodontics, Shanghai Ninth People’s Hospital, Shanghai Jiao Tong University School of Medicine, College of Stomatology, Shanghai Jiao Tong University, Shanghai 200011, China; luminous@sjtu.edu.cn (J.G.); zincomin@sjtu.edu.cn (W.Y.); superzhang@sjtu.edu.cn (Y.Z.); guxy1236@sh9hospital.org.cn (X.G.); 2 National Center for Stomatology & National Clinical Research Center for Oral Diseases & Shanghai Key Laboratory of Stomatology & Shanghai Research Institute of Stomatology, Shanghai 200011, China; 324015@sh9hospital.org.cn (L.Z.); luoluo20090214@sjtu.edu.cn (F.H.); xuyw3073@sh9hospital.org.cn (Y.X.); 3 Department of Dental Technology, Shanghai Ninth People’s Hospital, Shanghai Jiao Tong University School of Medicine, College of Stomatology, Shanghai Jiao Tong University, Shanghai 200011, China; 4 Dental Materials Science, Applied Oral Sciences and Community Dental Care, Faculty of Dentistry, The University of Hong Kong, Hong Kong SAR, China

**Keywords:** micro-computed tomography, desktop scanner, digital impression, digital dentistry, accuracy

## Abstract

**Background/Objectives:** This study aimed to evaluate the clinical feasibility of a novel desktop micro-computed tomography (micro-CT) scanner for digital impressions through comprehensively assessing its dimensional trueness and morphological accuracy in comparison with other optical-based scanners. **Methods**: A modified reference model was used to create ten silicone impressions and corresponding plaster models. Four digitization protocols were evaluated: (1) direct scanning of impressions via micro-CT scanner (MCTI), (2) extraoral scanning of impressions via F8 scanner (F8I), (3) extraoral scanning of plaster models via F8 scanner (F8PM), and (4) intraoral scanning of plaster models using Trios 5 scanner (IOSPM). Dimensional trueness was quantified via six linear measurements, and morphological accuracy (trueness and precision) was assessed by 3D surface deviation analysis. **Results**: Statistically significant differences in linear measurements between the digital impressions and the reference model were observed (*p* < 0.05). MCTI, F8PM and IOSPM demonstrated higher dimensional trueness than F8I. Although all methods showed high morphological precision, F8I (398.5 ± 43.0 µm) exhibited significantly greater root mean square (RMS) deviations for morphological trueness than MCTI (114.8 ± 42.2 µm), F8PM (142.1 ± 27.7 µm) and IOSPM (134.6 ± 12.0 µm) (*p* < 0.01). MCTI also demonstrated the highest reliability for morphological trueness according to relative standard deviation (RSD) analysis, with RSD values of 30.83% for MCTI, 41.80% for F8I, 37.26% for F8PM, and 42.55% for IOSPM. **Conclusions**: The novel micro-CT scanner enables accurate and reliable direct digitization of dental impressions. Its performance is comparable to scanning plaster models with high-end scanners and significantly superior to direct optical scanning of impressions, making it a promising alternative in digital dental workflow.

## 1. Introduction

Three-dimensional surface morphological data of the patient’s teeth and tissue in the oral cavity are obtained through intraoral and extraoral scanners, which are referred to as digital impressions [1,2,3]. Based on the digital data, the technology of computer-aided design and computer-aided manufacturing (CAD-CAM) has enabled the production of dental prostheses with higher efficiency and accuracy compared to traditional methods [4].

Digital impressions are typically generated either by scanning a plaster model produced through conventional impression techniques or by direct intraoral scanning using an intraoral scanner (IOS) [5]. However, each of these methods has inherent limitations. Extraoral scanning of plaster model still necessitates the fabrication of a physical stone cast, which introduces the risk of geometric inaccuracies due to manual handling and prolonged storage [6,7]. Additionally, this approach does not result in material conservation. On the other hand, while intraoral scanners offer advantages such as simplified workflow and reduced material usage compared to extraoral methods, their accuracy remains highly operator-dependent, influenced by the clinician’s experience and scanning technique [8,9,10]. Moreover, the accuracy of intraoral scanning can be compromised by various clinical factors, such as restricted mouth opening, involuntary tongue movements and the presence of oral moisture [11].

To overcome the limitations of both plaster model and intraoral scanning, impression scanning has been investigated as an alternative. However, the reported outcomes have been inconsistent. Shen [12] found that optical scanning of impression yielded accuracy comparable to that of plaster model scanning. In contrast, Kerr [13] observed that the optical scanner were unable to effectively capture undercut regions in the impressions. The limitations in capturing undercut regions can be partly attributed to the working principle of both extraoral and intraoral scanners, which rely on the acquisition and stitching of optical images [14,15], making their performance susceptible to various scanning conditions. Notably, inconsistent light reflection, particularly on steep surfaces or undercuts with sharp curvature changes, can hinder accurate surface capture, leading to deviations in the scan data [16,17]. These optical challenges ultimately restrict the feasibility of direct scanning impressions using optical scanners. CT-based scanning method offers an alternative approach for digitizing dental impressions by measuring the electron density of an object through the attenuation coefficient of the X-ray beams, which is captured by a detector array [18]. Compared to optical scanning, this method is unaffected by surface reflectivity. Indeed, in terms of precision metrology, metrological X-ray CT is commonly used in coordinate measuring systems (CMS) [19]. While the accuracy of cone beam computed tomography (CBCT) has been explored in several studies [20,21,22,23], its clinical application remains limited due to the need for complex post-processing, manually reconstruction, and the low resolution of the resulting digital dental casts. These factors ultimately reduce its efficiency and feasibility in dental practice.

Despite the advantages and limitations of existing scanners, a more precise and efficient approach to obtain digital impressions remains necessary. A newly introduced desktop micro-CT dental scanner presents a promising alternative by enabling automated, high-resolution reconstruction of conventional dental impressions. Unlike optical scanners, which rely on surface reflection and may be affected by undercuts, moisture and material texture, micro-CT can capture a series of X-ray images from 360-degree angles and reconstruct the dental model through advanced computational processing. Micro-CT typically offers higher resolution and a more sensitive detector than CBCT, enabling clearer visualization of fine anatomical structures [24]. Accordingly, micro-CT has been widely adopted in dental research as a reliable tool for high-fidelity 3D measurement, particularly for assessing the marginal and internal fit of dental restorations [25,26,27]. Previous studies by Kerr [13] and Kim [28] have further demonstrated the high accuracy of micro-CT in digitizing dental impressions, supporting its potential as a reliable alternative to conventional optical-based scanning methods. Although micro-CT has been validated for dental impression scanning, most available studies have not simultaneously evaluated both dimensional and morphological accuracy under clinically relevant conditions. Moreover, the performance of the newly introduced desktop micro-CT scanner, which is designed to provide automated and clinic-ready solutions for impression digitization, has not been systematically evaluated. This gap highlights the need for a comprehensive assessment of its accuracy in dental impression digitization.

Therefore, the objective of this in vitro study was to compare and assess the dimensional trueness and morphological accuracy of digital impressions generated using four different scanning methods: direct scanning of impressions via desktop micro-CT dental scanner (MCTI), extraoral scanning of impressions via F8 desktop optical scanner (F8I), extraoral scanning of plaster models via F8 desktop optical scanner (F8PM), and intraoral scanning of plaster models using Trios 5 scanner (IOSPM). The F8 desktop scanner and the Trios 5 intraoral scanner were selected in this study due to their widespread adoption in clinical settings and established roles in representing two primary digital workflows: extraoral scanning and intraoral scanning. The null hypothesis postulated that there would be no significant differences in dimensional trueness and morphological accuracy among the different scanning methods.

## 2. Materials and Methods

### 2.1. Reference Model

**Figure 1:**
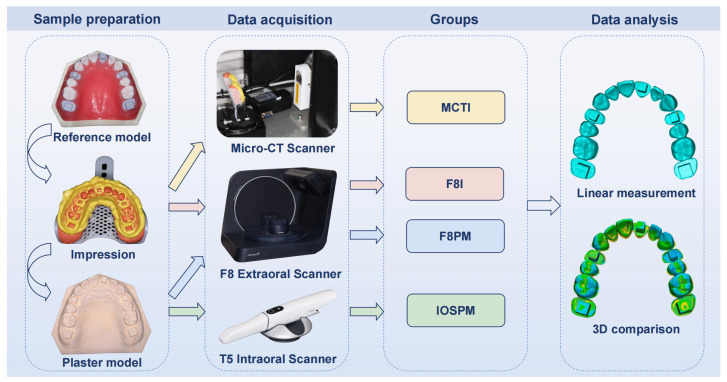
Workflow diagram of the study. Consistent color-coded arrows and icons are applied to distinguish the four scanning groups. The colors correspond to: yellow for MCTI group, red for F8I group, blue for F8PM group, and green for IOSPM group.

To assess the accuracy of digital impressions captured in conditions where external factors that may lead to errors, such as reflections due to saliva, are minimized, a Nissin plastic maxillary model(D16-500A(GSF)-QF, Nissin Dental, Kyoto, Japan) served as the reference in this study. For the purpose of linear distance measurements, five rectangular tooth phantoms were designed using engineering software (Geomagic Studio 2013, 3D Systems, Rock Hill, SC, USA) and fabricated with a 3D printer (UltraCraft A2D, HeyGears, Guangzhou, China). The phantoms replaced teeth at positions #17, #14, #21, #24 and #27 (Figure 1), with dimensions of 4 mm × 4 mm × 5 mm for positions #14, #21 and #24, and 6 mm × 6 mm × 5 mm for positions #17 and #27. Prior to use, all phantoms were dimensionally verified using a digital caliper (500-196-20 Digimatic Caliper, Mitutoyo, Kanagawa, Japan) to ensure measurement accuracy and reproducibility.

### 2.2. Sample Preparation

The sample size was determined using statistical software (PASS 15.0, NCSS, LLC, Kaysville, UT, USA). A pilot experiment was conducted to obtain preliminary means and standard deviations of the linear distance measurements across all groups. Sample size calculation was performed using a one-way analysis of variance (ANOVA) with a statistical power of 0.9 and a significance level of 0.05. Based on the pilot data, a large effect size of 0.9481 was obtained, indicating that at least six specimens per group were required. Considering both the calculated requirement and the sample sizes reported in similar studies [22,29], a total of ten specimens per group was selected to ensure a more robust and conservative study design.

A total of 10 impressions and plaster models were prepared following the manufacturers’ instructions. Initially, impressions of the reference model were taken using plastic trays and vinyl polysiloxane impression material (Express STD and Imprint II Garant Light Body, 3M ESPE, Neuss, Germany). After scanning the impressions, the corresponding plaster models were fabricated using Type 4 dental stone (Marmoplast N, Siladent, Goslar, Germany).

### 2.3. Scanning Methods

Based on the different scanning methods, four distinct groups were categorized: MCTI, F8I, F8PM, and IOSPM (Figure 1). All scanners underwent full manufacturer-recommended calibration prior to the experiment, and their calibration status remained stable throughout all scanning procedures. Each scanning procedure was repeated three times per sample to minimize potential errors. In total, 120 digital impressions were acquired and saved in standard tessellation language (STL) format for further analysis.

#### 2.3.1. Direct Scanning of Impressions Using Desktop Micro-CT Dental Scanner (MCTI)

The new desktop micro-CT dental scanner (Desktop Scanner, TDMIC-CT090, iRay Technology, Shanghai, China) was equipped with a CMOS detector, a rotating platform, and a radiation source operating at a voltage of 90 kV and a current of 88 µA. After the acquisition of dental impressions, the impressions were positioned vertically on the rotating platform and exposed to X-ray radiation in the chamber of the micro-CT dental scanner. As X-rays passed through the sample, two-dimensional projection images were generated from multiple angles during a 360-degree rotation. All scans were reconstructed with a voxel size of 10 µm. Following standardized post-processing of the reconstructed volumetric data, the digital impressions were automatically generated.

#### 2.3.2. Extraoral Scanning of Impressions (F8I) and Plaster Models (F8PM) Using F8 Desktop Optical Scanner

Simultaneously, the impressions were scanned using the 3Shape F8 laboratory scanner (F8, 3Shape, Copenhagen, Denmark), with the tray handle securely fixed on the impression fixture and the height adapter. Given the limitations reported in previous research [13], intraoral scanning of impressions was excluded from the present study due to incomplete data and the presence of distorted features in the scans. Following the scanning of impressions, plaster models were fabricated following the manufacturer’s instructions and scanned using the F8 laboratory scanner.

#### 2.3.3. Intraoral Scanning of Plaster Models Using Trios 5 Scanner (IOSPM)

The plaster models were also scanned using the wireless intraoral scanning device (TRIOS 5, 3Shape, Copenhagen, Denmark). The manufacturer’s recommended protocol for IOS usage was strictly followed, as outlined below: the scanner head was positioned on the occlusal surface of the last molar and slowly moved towards the anterior teeth, continuing until reaching the last molar on the opposite side. The scanner head was gently tilted to either side of the central axis to ensure comprehensive data capture. The head was then rotated to scan the palatal surfaces, before being turned back to scan the buccal surfaces, thereby generating the IOS-based digital impressions.

### 2.4. Linear Distance Measurement

The Mesial-Buccal-Occlusal points of five phantoms were selected to define six linear distances representing short, medium, and long spans (Table 1). All linear distance measurements were performed using Imageware software (Imageware 13.2, Siemens Digital Industries Software, Plano, TX, USA). To ensure measurement reliability, each sample was scanned three times, and for each scan, the corresponding STL file was measured three times for each linear distance, resulting in a total of nine measurements per linear distance for each sample. The final value for each linear distance of each sample was calculated as the mean of these nine individual measurements. All linear measurements were performed by a single trained operator. Intra-operator reliability was evaluated using the intraclass correlation coefficient (ICC). A two-way mixed model with absolute agreement was applied using SPSS software (IBM SPSS Statistics 26.0, IBM, New York, NY, USA) to assess measurement consistency.

For comparison, the control group DCRM consisted of linear distance measurements obtained from the reference model using a digital caliper (500-196-20 Digimatic Caliper, Mitutoyo, Kanagawa, Japan), with each linear distance measured nine times. The final linear distance was determined as the mean of nine measurements and established as the gold standard.

Dimensional trueness was assessed at two levels: between-group trueness was evaluated by comparing the linear distances among four test groups, whereas within-group trueness was determined by comparing the linear distances of each test group to the gold standard values.

### 2.5. Three-Dimensional Surface Deviation Analysis

**Figure 2:**
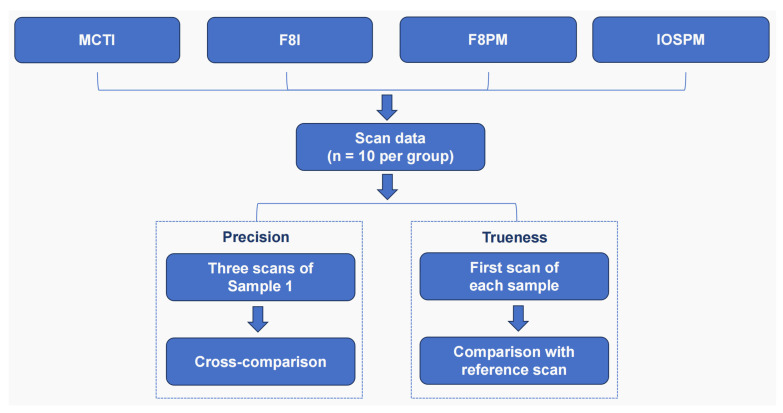
Flowchart for morphological precision and trueness evaluation of digital impressions.

To establish the reference dataset for 3D surface deviation analysis, the reference model was scanned using a high-resolution tabletop 3D dental scanner (AICON Breuckmann d-STATION-R1, Hexagon, Stockholm, Sweden). This scanner has been recognized for its high accuracy in digitizing small-scale objects and its consistent ability to capture detailed and reliable surface morphology of dental models. The Breuckmann reference file, together with the datasets obtained from MCTI, F8I, F8PM and IOSPM, were imported into 3D inspection and metrology software (Geomagic Control X 2020, 3D Systems, Rock Hill, SC, USA). To minimize errors associated with adjacent soft tissue, regions including the gingival margin were removed using a 3D modeling tool (Geomagic Studio 2013, 3D Systems, Rock Hill, SC, USA), enabling the superimposition of the clinical crowns and phantoms. After performing optimal best-fit alignment, 3D surface deviation analysis was conducted to assess the morphological accuracy (precision and trueness) [30]. In order to evaluate the precision of the scanners, the three scan files obtained from Sample 1 in each group were cross-compared (Figure 2). Morphological trueness of the digital impressions was evaluated by comparing the first scan of each sample in all test groups with the reference scan (Figure 2). Three-dimensional surface deviation analysis involved the calculation of the root mean square (RMS) of the deviations at each measurement point, with the RMS value representing the overall deviation between two scans for both precision and trueness [31]. In addition to RMS value for morphological trueness, the mean, standard deviation (SD) and relative standard deviation (RSD) of deviations at each measurement point were reported. RSD, calculated as (SD/mean) × 100%, provided a normalized measure of variability and enabled assessment of reliability of different scanning methods. Lower RSD values reflected higher reliability.

### 2.6. Statistical Analysis

In SPSS software (IBM SPSS Statistics 26.0, IBM, New York, NY, USA), the normality of the data distribution was assessed using the Shapiro–Wilk test. One-way analysis of variance (ANOVA) was performed to compare discrepancies in linear distances, as well as RMS values and mean deviations derived from 3D surface deviation analysis, among digital impressions generated by different scanning methods. In addition, one-sample *t*-test was conducted to evaluate the deviations in linear distances between the digital impressions and the ideal reference values from DCRM in each group. A significance level of α = 0.05 was set for all statistical analyses.

## 3. Results

### 3.1. Linear Distance Measurement

The results of the linear distance measurements were summarized in Table 2. The intra-operator reliability was excellent across all groups, with ICC values of 0.937 for MCTI, 0.986 for F8I, 0.955 for F8PM, and 0.951 for IOSPM. No statistically significant difference was observed in the linear distance discrepancy among the MCTI, F8PM, and IOSPM methods (*p* > 0.05), except for segment B in the F8PM group (*p* < 0.01) (Table 2). However, the discrepancy in linear distances between the F8I and the other test groups was statistically significant (*p* < 0.01). One-sample *t*-tests revealed statistically significant differences in four linear distances for both the MCTI and IOSPM groups (*p* < 0.05), when compared with the good standard values from DCRM. In addition, all six linear distances showed statistically significant differences in the F8PM (*p* < 0.05) and F8I groups (*p* < 0.01) (Table 3). For easier interpretation, the absolute deviations from the gold standard for each group are also provided in Table A1.

### 3.2. Three-Dimensional Surface Deviation Analysis

**Figure 3:**
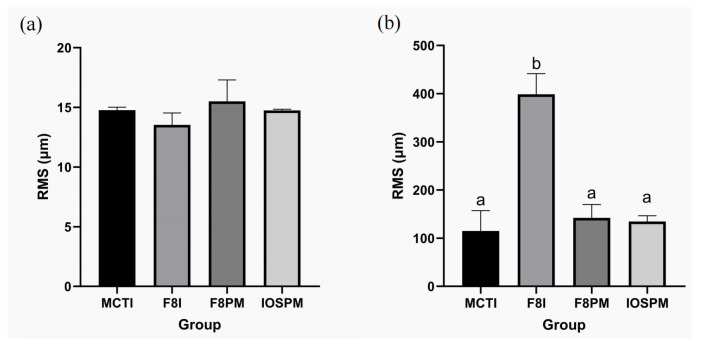
Bar charts of RMS values for morphological precision and trueness. (**a**) The RMS value for morphological precision of the digital impressions was analyzed using one-way ANOVA. No statistically significant difference was found among the groups (*p* = 0.219). (**b**) The RMS value for morphological trueness of the digital impressions was analyzed using one-way ANOVA. Different letters denote a statistically significant difference between groups (*p* < 0.01), while identical letters indicate no significant difference (*p* > 0.05).

**Figure 4:**
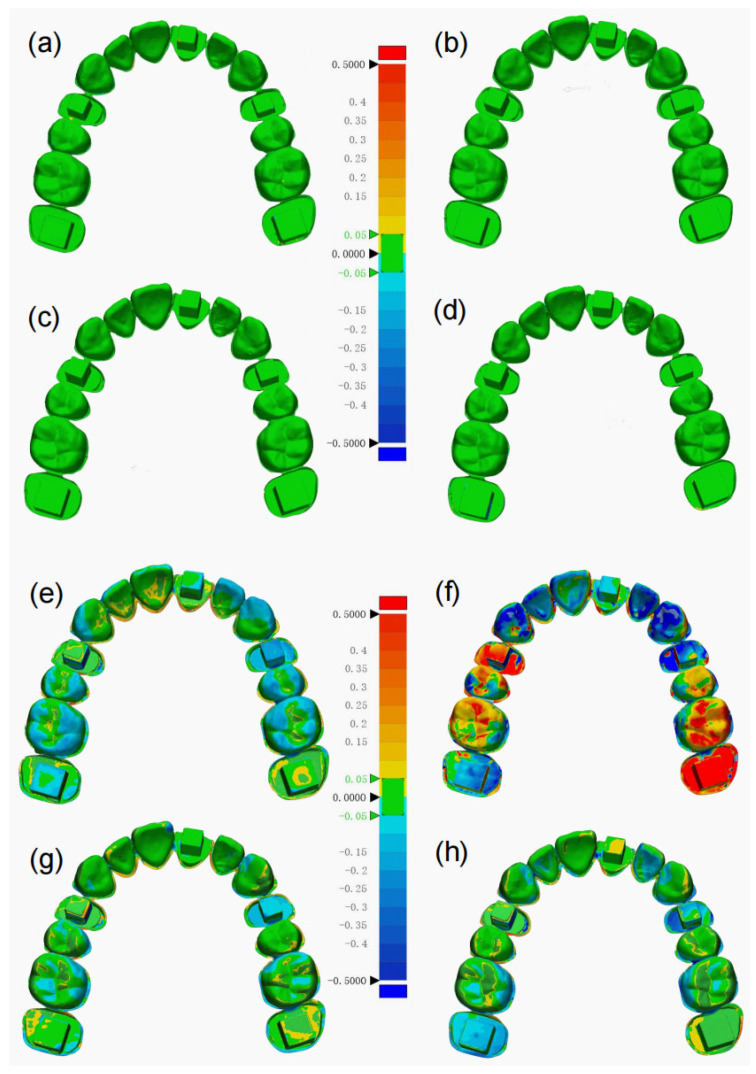
Three-dimensional surface deviation analysis color maps of morphological precision and trueness. (**a**–**d**) Representative color maps showing morphological precision of the digital impressions for the four groups: (**a**) MCTI, (**b**) F8I, (**c**) F8PM, (**d**) IOSPM. (**e**–**h**) Representative color maps showing morphological trueness of the digital impressions with respect to the reference scan. (**e**) MCTI, (**f**) F8I, (**g**) F8PM, (**h**) IOSPM.

The RMS values reflecting morphological precision of MCTI (14.8 ± 0.3 µm), F8I (13.5 ± 1.0 µm), F8PM (15.5 ± 1.8 µm) and IOSPM (14.7 ± 0.1 µm) methods showed no statistically significant difference among the groups (*p* = 0.219), as assessed by the one-way ANOVA (Figure 3 and Figure 4). In terms of morphological trueness, the RMS values of the MCTI (114.8 ± 42.2 µm), F8PM (142.1 ± 27.7 µm) and IOSPM (134.6 ± 12.0 µm) groups were notably lower than that of the F8I group (398.5 ± 43.0 µm). One-way ANOVA revealed statistically significant differences between the F8I group and the other three groups (*p* < 0.01) (Figure 3 and Figure 4), while no statistically significant differences were found among MCTI, F8PM, and IOSPM (*p* > 0.05). Similarly, for mean deviations, MCTI (11.47 ± 3.5365 µm), F8PM (14.15 ± 5.2726 µm) and IOSPM (13.87 ± 5.9018 µm) groups also outperformed F8I group (41.87 ± 17.5027 µm). One-way ANOVA identified significant differences in mean deviations between F8I and the other groups (*p* < 0.01), while no significant differences were observed among MCTI, F8PM and IOSPM (*p* > 0.05). In addition, the RSDs were calculated to assess the reliability of each scanning methods: 30.83% for MCTI, 41.80% for F8I, 37.26% F8PM, and 42.55% for IOSPM, with MCTI demonstrating the highest reliability.

## 4. Discussion

As digital workflows are increasingly integrated into dentistry, obtaining accurate virtual 3D models has become crucial. These digital workflows offer numerous advantages, including enhanced efficiency, streamlined data storage and improved reproducibility, all of which contribute to the development of advanced treatment protocols. However, the rapid introduction of new technological advancements and equipment has made it challenging for clinicians to select the most suitable method. To address this challenge, the present study seeks to evaluate the accuracy of a novel micro-CT scanning technique, alongside two commonly used scanning methods: F8 desktop optical scanner and Trios 5 intraoral scanner.

Accuracy is determined by both trueness (systematic error) and precision (random error). Trueness refers to the closeness of agreement between the test values and the real values, while precision describes the consistency of results obtained from repeated scans using the same scanner [32,33,34]. Together, these two factors define the overall accuracy of the experimental device. In the present study, both of systematic and random errors were evaluated.

Most studies evaluating the trueness of digital impressions have focused either on dimensional trueness or morphological trueness as key evaluation metrics [21,29,35]. Dimensional trueness typically refers to the linear distance discrepancies between digital impressions and plaster models, with the later serving as the reference model measured using a Vernier caliper [20,21]. Morphological trueness, on the other hand, is commonly assessed through 3D surface deviation analysis, which quantifies the morphological discrepancies between digital impressions and a reference model [36,37,38]. Some studies have also evaluated morphological precision by performing 3D surface deviation analysis between digital impressions obtained from the same scanning method [30,39]. The aforementioned evaluation methods were integrated and optimized to conduct this study. Accuracy was evaluated in both dimensional and morphological aspects, with the reference model serving as the gold standard. Based on the results, the null hypothesis stating that there were no significant differences in dimensional and morphological trueness among the different scanning methods was rejected. However, the hypothesis of no significant differences in morphological precision within each scanning method was retained.

Dimensional trueness was assessed by comparing linear distance discrepancies between the digital impressions and the reference model. Regardless of whether the discrepancies were positive or negative, the findings were consistent with similar studies [13,20]. The linear distance measurements obtained from the MCTI and IOSPM groups were closer to the gold standard compared to the other two groups. The F8I group, in particular, exhibited significantly lower linear distance measurements than the other groups and the gold standard. This discrepancy was primarily attributed to the F8 scanner’s inability to effectively project light into and capture reflections from the internal base of the silicone impressions, resulting in greater deviations between the digital impressions and the reference model. The linear distance measurements of the F8PM group were slightly higher than those of the MCTI, IOSPM groups and the gold standard. This deviation may be ascribed to the scanner’s limited ability to capture the vertices of rectangular surfaces on plaster models. To compensate for this limitation, the desktop optical scanner applied optimization algorithms to enhance scan quality, which may have inadvertently introduced marginally increased discrepancies compared to the MCTI and IOSPM groups. This finding aligned with previous research [36], which reported increased discrepancies in occlusal surface scans using desktop optical scanner. Another potential confounding factor is the scanner calibration status. All scanners were calibrated in accordance with manufacturer recommendations prior to the scanning procedures. However, calibration was not repeated between individual scans, which may have introduced minor variability and should be considered when interpreting the results. Although statistically significant differences in dimensional trueness were identified between the experimental groups and the gold standard, the deviations observed in the MCTI, F8PM and IOSPM groups remained within clinically acceptable thresholds [20]. Additionally, linear distance measurements across different segment lengths demonstrated comparable accuracy, suggesting that scan span had no significant effect on the performance of scanners. Therefore, these findings supported the clinical applicability of MCTI, F8PM, and IOSPM scanning methods for full-arch digital impression procedures.

In terms of morphological precision, excellent performance was observed across all groups, which is consistent with findings from similar studies [16,30]. The RMS values of the digital impressions indicated that the MCTI (114.8 ± 42.2 µm), F8PM (142.1 ± 27.7 µm), and IOSPM (134.6 ± 12.0 µm) groups exhibited significantly greater morphological trueness compared to the F8I group (398.5 ± 43.0 µm). This trend aligned with the findings from the linear distance measurements, further supporting the superior performance of the MCTI, F8PM, and IOSPM groups in maintaining trueness of the digital impressions. The color histogram revealed that deviations were predominantly observed in the posterior regions, consistent with the previous study [11]. The intricate morphology of the cusps, fossae, grooves and ridges in the posterior regions presented challenges for accurate light reflection capture and model reconstruction, resulting in varying degrees of positive and negative biases across different methods. Notably, the failure of the F8I group to effectively project light and capture reflections was clearly illustrated in the deviation map, where large areas of pronounced deviation were visualized, highlighting its limitations in accurately digitizing the internal surfaces of the silicone impressions. Therefore, this method is strongly discouraged for use in dental practice. The observed accuracy was inevitably lower than the theoretical ideal (i.e., zero deviation), due to the accumulation of potential errors introduced during the impression-taking, plaster model fabrication, image registration, and scanning procedures [40]. Nevertheless, a discrepancy range of 0.15 to 0.20 mm has been deemed acceptable in previous studies [20,22]. Accordingly, MCTI, F8PM and IOSPM groups still fulfilled the clinical requirements. Importantly, MCTI group demonstrated the lowest numerical deviation, reflecting superior morphological trueness compared to F8PM and IOSPM. In addition, the RSD analysis demonstrated that MCTI (30.83%) exhibited greater reliability than F8I (41.80%), F8PM (37.26%) and IOSPM (42.55%). This difference may primarily be attributed to the distinct working principles of the micro-CT scanner and optical scanners. Previous studies [25,26,27] have consistently demonstrated the methodological robustness of micro-CT, establishing it as a reliable tool for high-fidelity three-dimensional measurements in dental research.

Beyond restorative and prosthetic applications, micro-CT has been increasingly employed in other dental research fields, including endodontics, for detailed analysis of canal morphology, disinfection efficacy, and fluid dynamics. This trend highlights the versatility of micro-CT as a high-resolution tool for both structural and functional studies. Recent work by Puleio et al. [41] demonstrated the use of tomographic imaging to assess vapor lock effects on endodontic disinfection, confirming the potential of CT-based imaging for advanced evaluation of root canal procedures.

While MCTI, F8PM and IOSPM all achieved high dimensional and morphological accuracy, MCTI exhibited superior reliability, likely attributable to its imaging principle being less affected by external factors. Based on X-ray penetration imaging, micro-CT does not rely on surface optical reflection. This allows stable and complete acquisition of complex three-dimensional structures, including undercuts, without being affected by surface gloss, texture or shape of the object [42]. In contrast, the reduced reliability observed in plaster scanning may also stem from incomplete flow of liquid plaster into the impression surfaces, leading to voids around margins, undercuts and pinholes that compromised scan consistency [43]. Furthermore, the marginally lower reliability associated with intraoral scanning may be attributed to operator-dependent factors and inherent procedural inconsistencies [9,44]. In clinical settings, additional challenges such as saliva, variable reflection indices, and patient movement may further diminish the accuracy of intraoral scanning [45]. Notably, previous studies have reported that silicone impressions for implant restorations can achieve superior accuracy compared with intraoral scans [46]. This finding suggests that desktop micro-CT-based digitization may offer a reliable digital workflow for implant impression processing.

Taken together, the novel desktop micro-CT dental scanner demonstrated superior accuracy and reliability, supporting its clinical applicability as a promising alternative to conventional optical and plaster-based scanning workflows. By enabling direct digitization of dental impressions into high-accuracy digital models, this approach reduces potential errors and time consumption associated with plaster model fabrication. Clinically, it allows dental practitioners to seamlessly integrate traditional impression techniques with digital workflows, thereby enhancing treatment precision and efficiency.

However, this study had several limitations. First, manufacturer-recommended scanner calibration was performed prior to use, but repeated calibration between scans was not conducted, which may slightly introduce measurement variability. Second, this study focused exclusively on dimensional and morphological accuracy, without considering qualitative characteristics of impressions or plaster models. In addition, as an in vitro study, the use of plaster models as the research object for the intraoral scanning group may limit the generalizability of these findings to real-world clinical settings. Nevertheless, the study was designed to obtain standardized measurements within a controlled environment, utilizing a maxillary typodont model as the reference, as it was impractical to use digital calipers on a live patient. Notwithstanding these limitations, the intraoral scanning group yielded results consistent with previous research [22], supporting the reliability of the findings. Future studies should address these limitations, implement repeated scanner calibration, and progress from in vitro experiments to in vivo studies to further validate these findings under real clinical conditions.

## 5. Conclusions

This study found that digital impressions obtained by direct scanning of impressions via micro-CT scanner (MCTI), extraoral scanning of plaster models via F8 scanner (F8PM), and intraoral scanning of plaster models using Trios 5 scanner (IOSPM) demonstrated superior accuracy compared with extraoral scanning of impressions via F8 scanner (F8I). Among these evaluated methods, MCTI achieved the lowest numerical deviation and the highest reliability in morphological trueness, highlighting its potential clinical advantage over other optical-based methods.

The novel desktop micro-CT scanner provides accurate and reliable digitization of dental impressions, demonstrating comparable or superior trueness to high-end optical and intraoral scanners.

## Figures and Tables

**Table 1:** Six linear distances measured.

Category	Linear Distance	Definition
Short Distance	Segment A	The distance between Mesial-Buccal-Occlusal points of #21 and #24
	Segment B	The distance between Mesial-Buccal-Occlusal points of #21 and #14
Medium Distance	Segment C	The distance between Mesial-Buccal-Occlusal points of #21 and #27
	Segment D	The distance between Mesial-Buccal-Occlusal points of #14 and #24
Long Distance	Segment E	The distance between Mesial-Buccal-Occlusal points of #17 and #27
	Segment F	The distance between Mesial-Buccal-Occlusal points of #14 and #27

# denotes the tooth number according to the FDI two-digit system.

**Table 2:** Discrepancy in linear distance measurements of digital impressions (Mean ± SD, mm).

Linear Distance (mm)	MCTI	F8I	F8PM	IOSPM
Segment A	23.7664 ± 0.0084 ^a^	23.4794 ± 0.0204 ^b^	23.7727 ± 0.0083 ^a^	23.7563 ± 0.0093 ^a^
Segment B	27.4392 ± 0.0066 ^a^	27.2171 ± 0.0061 ^b^	27.4604 ± 0.0041 ^c^	27.4338 ± 0.0068 ^a^
Segment C	46.8219 ± 0.0065 ^a^	46.5102 ± 0.0334 ^b^	46.8225 ± 0.0057 ^a^	46.8297 ± 0.0083 ^a^
Segment D	41.7104 ± 0.0061 ^a^	41.4567 ± 0.0271 ^b^	41.7224 ± 0.0048 ^a^	41.7242 ± 0.0063 ^a^
Segment E	54.2561 ± 0.0050 ^a^	54.0900 ± 0.0156 ^b^	54.2606 ± 0.0069 ^a^	54.2645 ± 0.0071 ^a^
Segment F	54.3134 ± 0.0086 ^a^	54.0960 ± 0.0275 ^b^	54.3131 ± 0.0038 ^a^	54.3231 ± 0.0077 ^a^

Different superscript lowercase letters (a, b, c) within the same row denote statistically significant differences between groups (*p* < 0.05), while groups sharing the same letter indicate no significant difference (*p* > 0.05). Results derived from one-way ANOVA.

**Table 3:** Statistical differences in linear distance discrepancies between each experimental group and gold standard values from DCRM.

Linear Distance (mm)	DCRM	MCTI		F8I		F8PM		IOSPM	
		t	*p*	t	*p*	t	*p*	t	*p*
Segment A	23.7656	0.291	0.778	−44.453	<0.001	2.677	0.025	−3.165	0.011
Segment B	27.4467	−3.644	0.005	−119.213	<0.001	10.579	<0.001	−6.039	<0.001
Segment C	46.8300	−3.965	0.003	−30.244	<0.001	−4.160	0.002	−0.122	0.905
Segment D	41.7189	−4.382	0.002	−30.607	<0.001	2.270	0.049	2.633	0.027
Segment E	54.2533	1.775	0.110	−33.098	<0.001	3.341	0.009	4.967	0.001
Segment F	54.3222	−3.235	0.010	−25.971	<0.001	−7.451	<0.001	0.367	0.722

Results from the one-sample *t*-test.

## Data Availability

The original contributions presented in this study are included in the article. Further inquiries can be directed to the corresponding authors.

## Abbreviations

The following abbreviations are used in this manuscript:

Micro-CT	Micro-computed tomography
MCTI	Direct scanning of impressions via desktop micro-CT dental scanner
F8I	Extraoral scanning of impressions via F8 desktop optical scanner
F8PM	Extraoral scanning of plaster models via F8 desktop optical scanner
IOSPM	Intraoral scanning of plaster models using Trios 5 scanner
DCRM	Linear distance measurements obtained from the reference model using a digital caliper
ICC	Intraclass Correlation Coefficient
RMS	Root mean square
RSD	Relative standard deviation
ANOVA	Analysis of variance
STL	Standard tessellation language

## Appendix A

**Table A1:** Absolute deviations of linear distance measurements of digital impressions from the reference model (Mean ± SD, mm).

Linear Distance (mm)	MCTI	F8I	F8PM	IOSPM
Segment A	0.0062 ± 0.0053	0.2862 ± 0.0204	0.0090 ± 0.0059	0.0099 ± 0.0087
Segment B	0.0079 ± 0.0060	0.2296 ± 0.0061	0.0137 ± 0.0041	0.0132 ± 0.0061
Segment C	0.0090 ± 0.0050	0.3198 ± 0.0334	0.0075 ± 0.0057	0.0063 ± 0.0050
Segment D	0.0085 ± 0.0060	0.2623 ± 0.0271	0.0045 ± 0.0038	0.0066 ± 0.0048
Segment E	0.0041 ± 0.0039	0.1633 ± 0.0156	0.0079 ± 0.0062	0.0112 ± 0.0071
Segment F	0.0104 ± 0.0064	0.2262 ± 0.0275	0.0091 ± 0.0038	0.0055 ± 0.0051
